# Transcriptomic Analysis of Gene Networks Regulated by U11 Small Nuclear RNA in Bladder Cancer

**DOI:** 10.3389/fgene.2021.695597

**Published:** 2021-07-02

**Authors:** Zhenxing Wang, Xi Wang, Yaobang Wang, Shaomei Tang, Chao Feng, Lixin Pan, Qinchen Lu, Yuting Tao, Yuanliang Xie, Qiuyan Wang, Zhong Tang

**Affiliations:** ^1^Center for Genomic and Personalized Medicine, Guangxi Medical University, Nanning, China; ^2^Guangxi Key Laboratory for Genomic and Personalized Medicine, Guangxi Collaborative Innovation Center for Genomic and Personalized Medicine, Nanning, China; ^3^Department of Clinical Laboratory, The First Affiliated Hospital of Guangxi Medical University, Nanning, China; ^4^Department of Gastroenterology, The First Affiliated Hospital of Guangxi Medical University, Nanning, China; ^5^Department of Urology, The Affiliated Cancer Hospital of Guangxi Medical University, Nanning, China; ^6^School of Information and Management, Guangxi Medical University, Nanning, China

**Keywords:** U11 small nuclear RNA, bladder cancer, T24, transcriptomic analysis, gene networks

## Abstract

Small nuclear RNA is a class of non-coding RNA that widely exist in the nucleus of eukaryotes. Accumulated evidences have shown that small nuclear RNAs are associated with the regulation of gene expression in various tumor types. To explore the gene expression changes and its potential effects mediated by U11 snRNA in bladder cancer cells, U11 snRNA knockout and overexpressed cell lines were constructed and further used to analyze the gene expression changes by RNA sequencing. The differentially expressed genes were found to be mainly enriched in tumor-related pathways both in the U11 knockout and overexpression cell lines, such as NF-kappa B signaling pathway, bladder cancer and PI3K-Akt signaling pathway. Furthermore, alternative splicing events were proposed to participate in the potential regulatory mechanism induced by the U11 knockout or overexpression. In conclusion, U11 may be involved in the regulation of gene expression in bladder cancer cells, which may provide a potentially new biomarker for clinical diagnosis and treatment of bladder cancer.

## Introduction

Bladder cancer is one of the most common urological cancers, ranking ninth among all malignant tumors worldwide and sixth among men (Ploeg et al., 2009; Bray et al., 2018). In the United States, bladder cancer ranks fourth among all malignant tumors, with 74,000 new cases of bladder cancer in 2015, including 56,320 males and 17,680 females, and the estimated fatal cases were 16,000, including 11,510 males and 4,490 females. In South Asia and Western Asia, the incidence and mortality of bladder cancer also rank top among all malignant tumors (Salim et al., 2010). Although the incidence and mortality of bladder cancer in China are lower than the world average level, there is a trend of increasing incidence in some cities (Feng et al., 2019), which seriously threatens the survival health and life quality of patients. Therefore, it is of great significance to study the mechanism of the occurrence and development of bladder cancer, and further to improve the diagnosis and treatment rate of bladder cancer.

Cajal bodies, also known as coiled bodies, were first discovered in the nucleus of nerve cells by Ramony Cajal in 1903 (Hebert, 2010). Cajal bodies are widely found in the nucleus of higher eukaryotes, and their number and size can vary with species. Cajal bodies are more abundant in somatic cells of differentiated tissues and some cells with higher metabolic activity, such as muscle, neurons, and tumor cells (Hearst et al., 2009; Machyna et al., 2013). At the same time, the numbers and sizes of Cajal bodies are related to the cell cycle, and their numbers often reach the maximum in the G1/S phase. In general, Cajal bodies depolymerize in the M phase, and the reassembly process depends on the level of gene transcription and the rate of proliferation of the cell. Relevant studies have shown that there are a large number of factors involved in the splicing of mRNA precursors, rRNA precursor processing, histone pre-mRNA 3′ end processing and telomere maintenance in the components of Cajal bodies, suggesting that Cajal bodies may be a site for the assembly and function of ribonucleoprotein (RNP) (Hebert, 2013). Cajal bodies can also serve as a platform for effective modification responses in transcriptionally active cells requiring high levels of RNP, such as neuronal cells and tumor cells. Studies have found that zebrafish embryos are unable to complete embryonic development due to the lack of coilin and Cajal bodies. The depletion of coilin and Cajal bodies was mainly characterized by deficits in snRNP biogenesis and expression of spliced mRNA, while mature snRNPs can partially rescue embryonic lethal phenotypes (Strzelecka et al., 2010).

At present, more than dozens of proteins have been found to localize or interact with Cajal bodies (Machyna et al., 2013). Coilin is recognized as a marker protein and a major component of Cajal bodies (Andrade et al., 1991). One of the most significant structural features of Cajal bodies is the accumulation of a large number of non-coding small RNAs, which include U1, U2, U3, U4, U5, U6, U7, U8, U11, U12, U13, U14, U64, U6atac, and U87 scaRNA, etc. These small non-coding RNAs were once thought to be mainly involved in post-transcriptional modification of RNA. However, with the development of high-depth sequencing technology and bioinformatics technology, the functions of these small non-coding RNAs have been further recognized and understood. Studies have found that they may be involved in gene expression, genome structure organization, and other functions (Lui and Lowe, 2013). In recent years, non-coding RNAs have attracted much attention as a special way of gene expression regulation.

Our previous studies innovatively proposed the notion that Cajal bodies can be simultaneously linked with multiple small molecule RNA gene loci to form gene clusters, using Hela cell models and six-color microscopes detection systems (Wang et al., 2016). Moreover, we found that this gene cluster is not formed randomly but is a specific spatial structure of long-distance interactions. U1, U2, U3, U4, U5, and U11 are small non-coding RNA genes enriched in Cajal bodies, U87 scaRNA is small Cajal body-specific RNA gene, Histone cluster 2 and Histone H3F3 are histone small RNA genes. Among them, U1, U2, U3, U4, U5, and U11 are small non-coding RNA genes enriched in Cajal bodies. Using chromatin spatial conformation capture technology, it was found that small nuclear RNA mediates the formation of long-distance chromatin interactions (Wang et al., 2016). U11 (RNU11) is probably the most significant small nuclear RNA because its expression is extremely high expressed in rapidly growing tumor cells and very low expressed in slow-growing primary cells. Thus, the above studies provided the evidence that U11 small nuclear RNA plays a role in regulating the spatial structure of chromatin and may be of great significance in the development of tumors.

Interestingly, we found Cajal bodies were aberrantly activated in T24 bladder cancer cell lines and the increased numbers and sizes of Cajal bodies were displayed in two highly invasive and metastatic T24-SLT and T24-FL cell lines. Given that U11 is one of the most crucial snRNAs located in Cajal bodies, we speculated that U11 might play an important role in the gene expression of bladder cancer cells. In this study, the *in vitro* cell models with U11 knockout and overexpression were firstly constructed in T24 bladder cancer cell lines and further used to analyze the gene expression changes using RNA-sequencing technology. The differentially expressed genes were found to be mainly enriched in tumor-related pathways both in the U11 knockout and overexpression groups. Notably, alternative splicing events were innovatively proposed to participate in the potential regulatory mechanism induced by U11 knockout or overexpression. Taken together, our study innovatively elucidated that U11 may play the critical role in the regulation of gene expression in bladder cancer cells, which may provide a potentially new biomarker for clinical diagnosis and treatment of bladder cancer.

## Results

### Cajal Body-Related snRNA U11 Affects the Occurrence and Development of Bladder Cancer by Regulating Differently Expressed Genes

**FIGURE 1:**
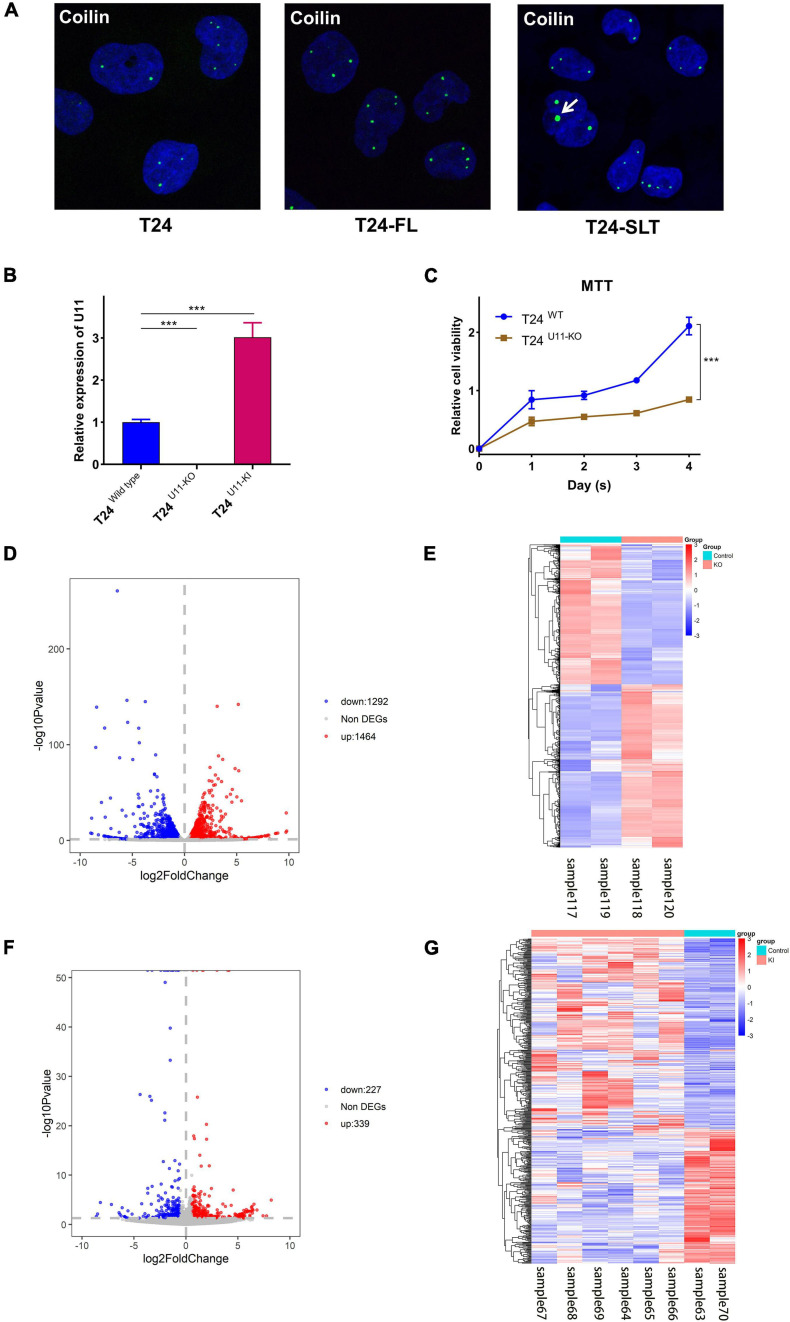
Construction of T24^U11–KO^ and T24^U11–KI^ cell lines and DEGs analysis of T24-related cell lines. **(A)** Immunofluorescence of Coilin in T24, T24-FT, T24-SLT cell lines. **(B)** The expression levels of U11 in T24^U11–KO^ and T24^WT^ cell lines. **(C)** Cell viability of T24^U11–KO^ and T24^WT^ cell lines. **(D)** Volcano plot for differentially expressed genes between T24^U11–KO^ and T24^WT^ cell lines. **(E)** Heatmap for differentially expressed genes between T24^U11–KO^ and T24^WT^ cell lines. **(F)** Volcano plot for differentially expressed genes of between T24^U11–KI^ and T24^WT^ cell lines. **(G)** Heatmap for differentially expressed genes of between T24^U11–KI^ and T24^WT^ cell lines. Differential expressed genes (DEGs): *P*-value < 0.05 and | Fold change| ≥ 1.5. ***P < 0.001.

**FIGURE 2:**
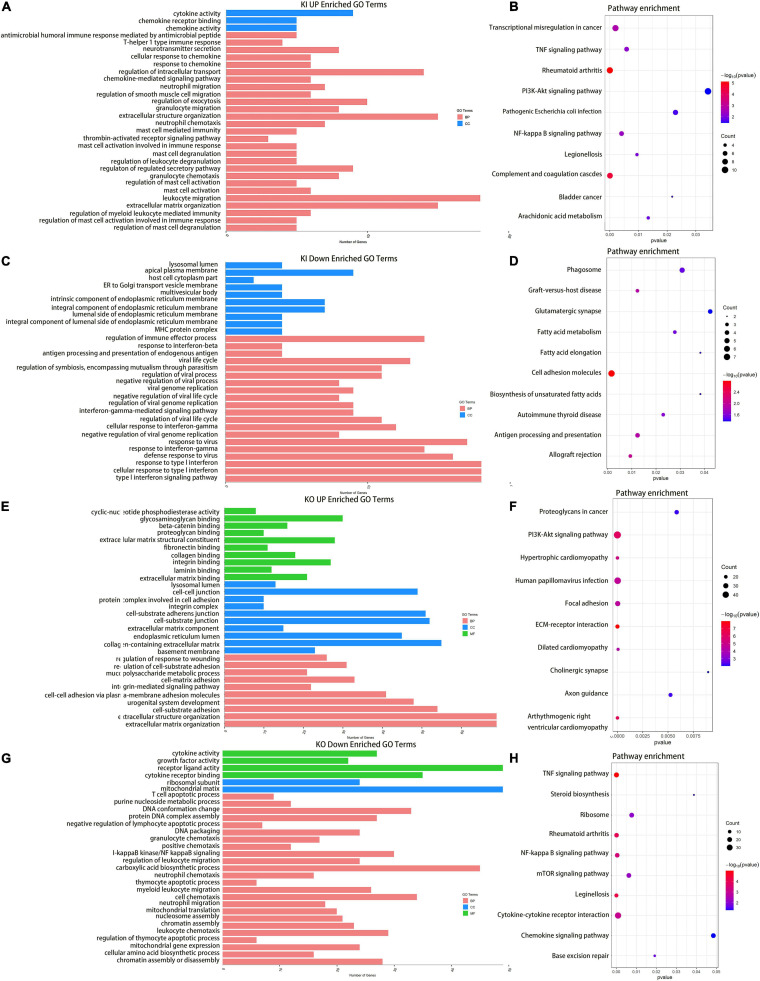
Enrichment Analysis for DEGs upon knocking out and overexpressing U11 in T24 cell lines. **(A,C)** GO pathways of upregulated and downregulated genes in T24^U11–KI^ cell line, respectively. **(B,D)** KEGG pathways of upregulated and downregulated genes in T24^U11–KI^ cell line, respectively. Panels **(E,G)** are GO pathways of upregulated and downregulated genes in T24^U11–KO^ cell line, respectively. Panels **(F,H)** are KEGG pathways of upregulated and downregulated genes in T24^U11–KO^ cell line, respectively. GO: gene ontology; KEGG: Kyoto Encyclopedia of Genes and Genomes.

**FIGURE 3:**
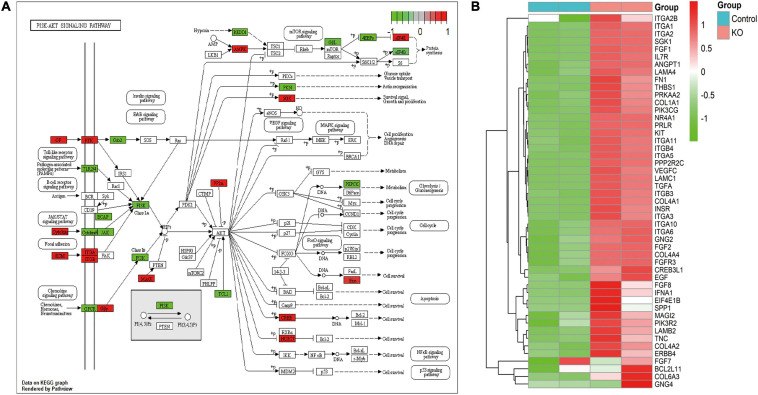
PI3K-Akt signaling pathway for DEGs from knocking out U11. **(A)** PI3K-Akt signaling pathway diagram. **(B)** Heatmap from DEGs in this pathway.

By using immunofluorescence (IF) staining, we unexpectedly found Cajal bodies were aberrantly activated in T24 bladder cancer cell lines. More interestingly, the increased numbers and sizes of Cajal bodies were displayed in two highly invasive and metastatic T24-SLT and T24-FL cell lines (Nicholson et al., 2004; Jeppesen et al., 2014) (Figure 1A). Given that U11 is one of the most crucial snRNAs located in Cajal bodies, the *in vitro* cell models with U11 knockout and overexpression were successfully established in T24 cell lines. The knockout and overexpression efficiency of U11 in T24 cell lines were confirmed by real-time quantitative PCR, the expression level of U11 in U11-KO cell line was significantly decreased, and that of U11-KI cell line was significantly overexpressed compared to T24 WT cell line (Figure 1B). Moreover, MTT assay revealed that the cell proliferation ability of T24 WT cell line was significantly higher than that of the U11-KO cell line (*P* < 0.001, Figure 1C). The U11 knockout and U11 overexpression groups were used as experimental groups, and gene differences were analyzed by comparing with the control group. A total of 2,756 differentially expressed genes in the U11 knockout group were obtained, including 1,464 up-regulated and 1,292 down-regulated (Figures 1D,E); In addition, there were 566 differentially expressed genes in the U11 overexpression group, including 339 up-regulated and 227 down-regulated (Figures 1F,G). Pathway enrichment analysis by clusterProfiler R package showed that the up-regulated genes obtained by overexpressing U11 were mainly enriched in regulation of mast cell degranulation, chemokine activity, NF-kappa B signaling pathway, TNF signaling pathway, and Bladder cancer, etc. (Figures 2A,B). The down-regulated genes obtained by overexpressing U11 were mainly enriched in integral component of lumenal side of endoplasmic reticulum membrane, cellular response to type I interferon, defense response to virus, Allograft rejection and Antigen processing and presentation, etc. (Figures 2C,D). Interestingly, we found that the enrichment pathways of down-regulated genes in the U11 knockout group were similar to those of up-regulated genes in the U11 overexpression group. The pathways of down-regulated genes in U11 knockout group were mainly enriched in T cell apoptotic process, cytokine receptor binding, TNF signaling pathway, and NF-kappa B signaling pathway, etc. (Figures 2G,H). The pathways of up-regulated genes in U11 knockout group were mainly enriched in laminin binding, fibronectin binding, cell-substrate adhesion, Proteoglycans in cancer, and PI3K-Akt signaling pathway (Figures 2E,F). Among them, Figure 3 showed the PI3K-Akt signaling pathway and the genes involved in this pathway.

**FIGURE 4:**
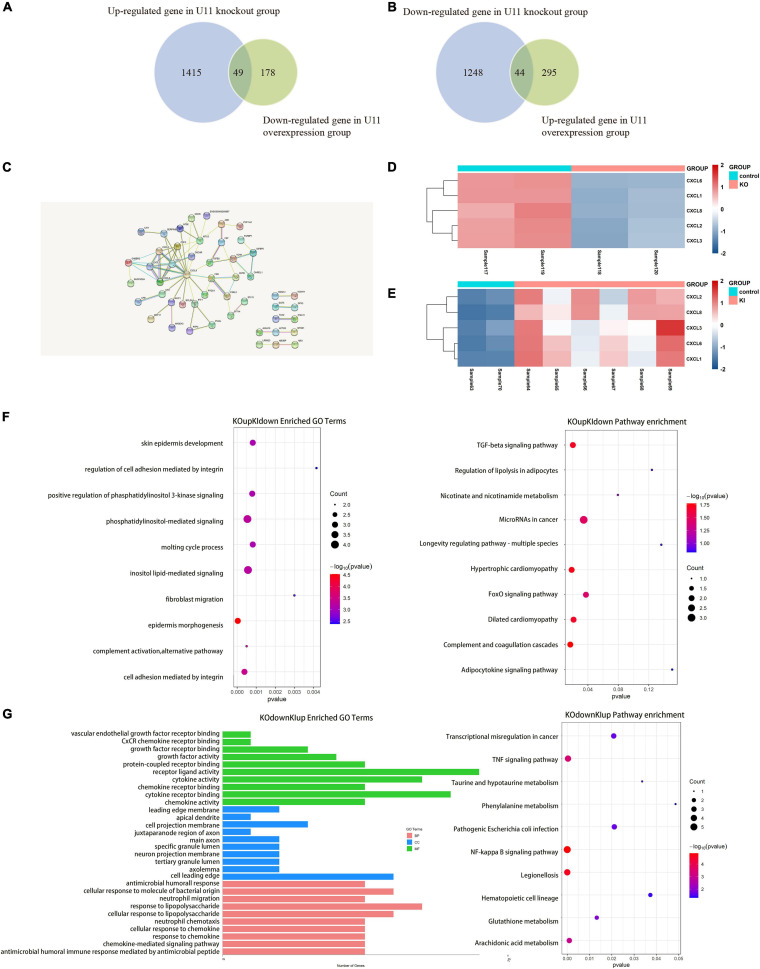
Comprehensive analysis of DEGs upon knocking out and overexpressing U11 in T24 cell lines. **(A,B)** Venn diagrams of the intersection of differentially expressed genes between U11 overexpression group and knockout group. **(C)** Protein–protein interactions (PPI) of intersection gene. **(D)** The heatmap of CXCL family genes in T24^U11–KO^ and T24^WT^ cell lines. **(E)** The heatmap of CXCL family genes in T24^U11–KI^ and T24^WT^ cell lines. **(F)** Pathways from intersecting genes of up-regulated genes in T24^U11–KO^ cell line and down-regulated genes in T24^U11–KI^ cell line. **(G)** Pathways from intersecting genes of down-regulated genes in T24^WT^ cell line and up-regulated genes in T24^U11–KI^ cell line.

Subsequently, we used the up-regulated genes in the U11 overexpression group and the down-regulated genes in the U11 knockout group for intersection analysis, as well as the down-regulated genes in the U11 overexpression group and the up-regulated genes in the U11 knockout group for intersection analysis. We found that there were 93 intersecting genes in the two intersecting groups (Figures 4A,B). Using these genes for protein interaction network analysis, we found that the hub genes were mainly CXCL2, CXCL3, CXCL6, CXCL8, and other CXCL gene families (Figure 4C). Interestingly, we found that the expression of CXCL2, CXCL3, CXCL6, CXCL8, and other CXCL gene families was significantly up-regulated in the U11 overexpression group, while the expression of these genes was significantly down-regulated in the U11 knockout group (Figures 4D,E). The intersecting genes of up-regulated genes in U11 knockout group and down-regulated genes in U11 overexpression group were mainly enriched in regulation of cell adhesion mediated by integrin, fibroblast migration and regulation of lipolysis in adipocytes (Figure 4F), and the intersecting genes of down-regulated genes in U11 knockout group and up-regulated genes in U11 overexpression group were mainly enriched in chemokine activity, response to lipopolysaccharide, neutrophil migration, NF-kappa B signaling pathway, TNF signaling pathway, and transcriptional mis-regulation in cancer (Figure 4G).

### U11 Alters Gene-Splicing Events and Gene Expression

**FIGURE 5:**
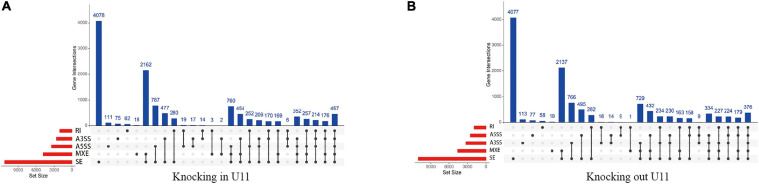
The identification of alternative splicing events. **(A,B)** Up-set plots of significant alternative splicing events upon knockout and overexpressed U11 in T24 cells. Skipped exon (SE), alternative 5′ splice site (A5SS), alternative 3′ splice site (A3SS), mutually exclusive exons (MXE), and retained intron (RI).

We further performed alternative splicing analysis and found that a total of 4,023 genes in the U11 overexpression group had significant differential alternative splicing events. Among them, exon skipping (SE) was the most frequent event, while intron retention (RI) was the least frequent event, and 316 genes were simultaneously exposed to five alternative splicing events (Figure 5A). Similarly, 4,774 genes were found to have significant differential alternative splicing events in the U11 knockout group, with exon skipping events occurring most frequently (Figure 5B).

**FIGURE 6:**
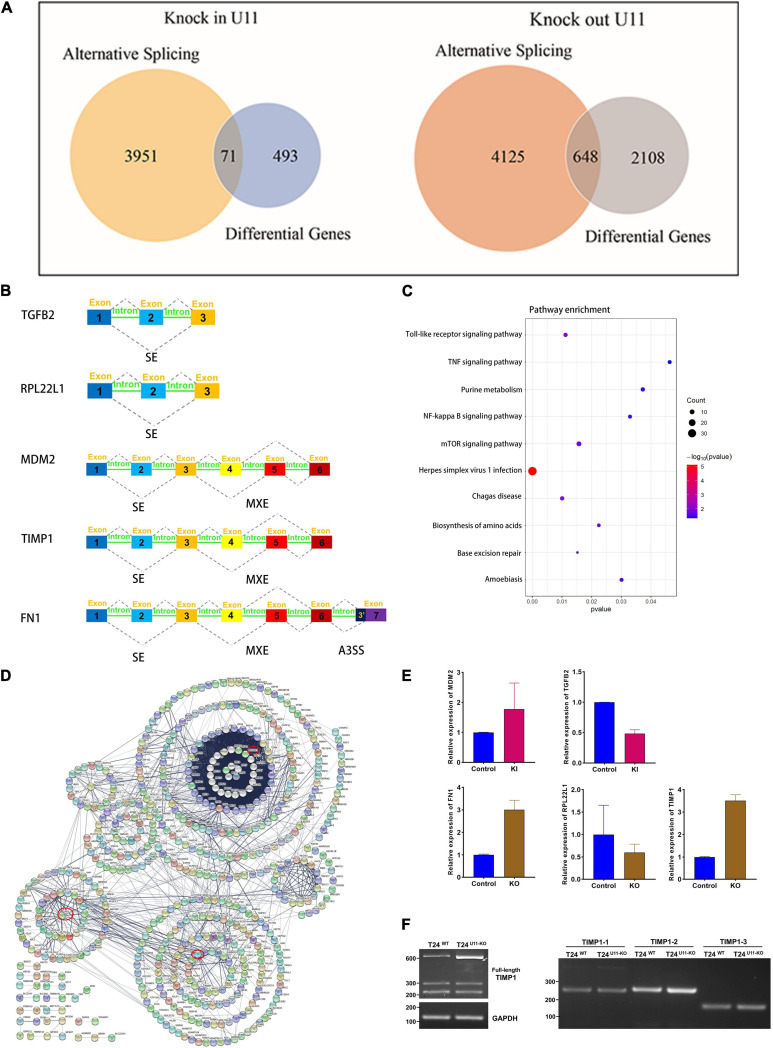
Comprehensive analysis of alternative splicing events and differential genes upon knocking out and overexpressing U11 in T24 cell lines. **(A)** Venn diagrams of DEGs and genes with significant alternative splicing events. **(B)** Diagrams of alternative splicing events in MDM2, TGFB2, RPL22L1, FN1, and TIMP1. Mutually exclusive exons (MXE) and skipped exon (SE) in MDM2; SE in TGFB2; SE in RPL22L1; MXE and SE in TIMP1; MXE, SE and alternative 3′ splice site (A3SS) in FN1. **(C)** Pathways from intersecting genes between T24^U11–KO^ and T24^WT^ cell lines. **(D)** Venn diagram of Protein–protein interactions (PPI) of intersecting genes. **(E)** The relative expression levels of MDM2 and TGFB2 between T24^U11–KI^ and T24^WT^ cell lines and the relative expression levels of FN1, RPL22L1, and TIMP1 between T24^U11–KO^ and T24^WT^ cell lines. **(F)** Alternative splice identification of TIMP1-full length, TIMP1-1, TIMP1-2, and TIMP1-3 in T24^U11–KO^ and T24^WT^ cell lines.

Next, we intersected genes with differentially alternative splicing events and differentially expressed genes (Figure 6A). Seventy-one intersecting genes were obtained in the U11 overexpression group. Among them, murine double minute 2 (MDM2) gene had one exon skip and one mutually exclusive exon event, and the gene expression increased about 3.5-fold, and TGFB2 gene had one exon skip, and the gene expression decreased 2.1-fold (Figures 6B,E). Notably, 648 intersecting genes in the U11 knockout group, which were mainly enriched in pathways such as NF-kappa B signaling pathway and TNF signaling pathway (Figure 6C). The results of protein interaction network analysis of these intersecting genes also showed that the hub genes mainly included genes such as TIMP1, FN1, and RPL22L1 (Figure 6D). Intriguingly, FN1 gene had multiple alternative 3′ splice site (A3SS) events, one mutually exclusive exon event, four exon skipping events, and the level of mRNA expression increased 2.9-fold. TIMP1 gene had only one exon skipping event, and the level of mRNA expression increased 3.5-fold. RPL22L1 gene had one exon skipping events, and the level of expression decreased 1.7-fold (Figures 6B,E).

Given that the alternative splicing events of TIMP1, FN1, and RPL22L1 have been widely reported to participate in several biological processes (Usher et al., 2007; Lopez-Mejia et al., 2013; Liang et al., 2019), we therefore validated the AS events of the genes described above, using PCR and gel electrophoresis. We initially examined three typical exons skipping of FN1 (EDA, EDB, and IIICS) as previously reported (Lopez-Mejia et al., 2013), but no remarkably alternative splice events were detected in FN1 gene. The full-length TIMP1 transcript was then detected by the forward primer located in exon 1 combined with the reverse primer located in exon 6. Intriguingly, the band of full-length TIMP1 in T24^U11–KO^ cell line was observed to shift down a weak distance less than an exon, compared with T24^WT^ cell line. What’ more, as the gel picture shown and the gray arrow indicated in Figure 6F, one indistinctly visible band appeared below the major band in T24^U11–KO^ cell line, but not in T24^WT^ cell line, suggesting a potential AS event of TIMP1 after U11 knockout. Three exons of TIMP1 gene were further examined the alternative splicing events, respectively. However, no significant alteration of splicing patterns was observed in the TIMP1-1, TIMP1-2, and TIMP1-3 segments because of the rare frequency and low abundance of the exon skipping (Figure 6F). All these results indicated that a link between alternative splicing regulated by U11 and bladder carcinogenesis.

### FN1 and RPL22L1 May Be a Prognostic Marker for Bladder Cancer

**FIGURE 7:**
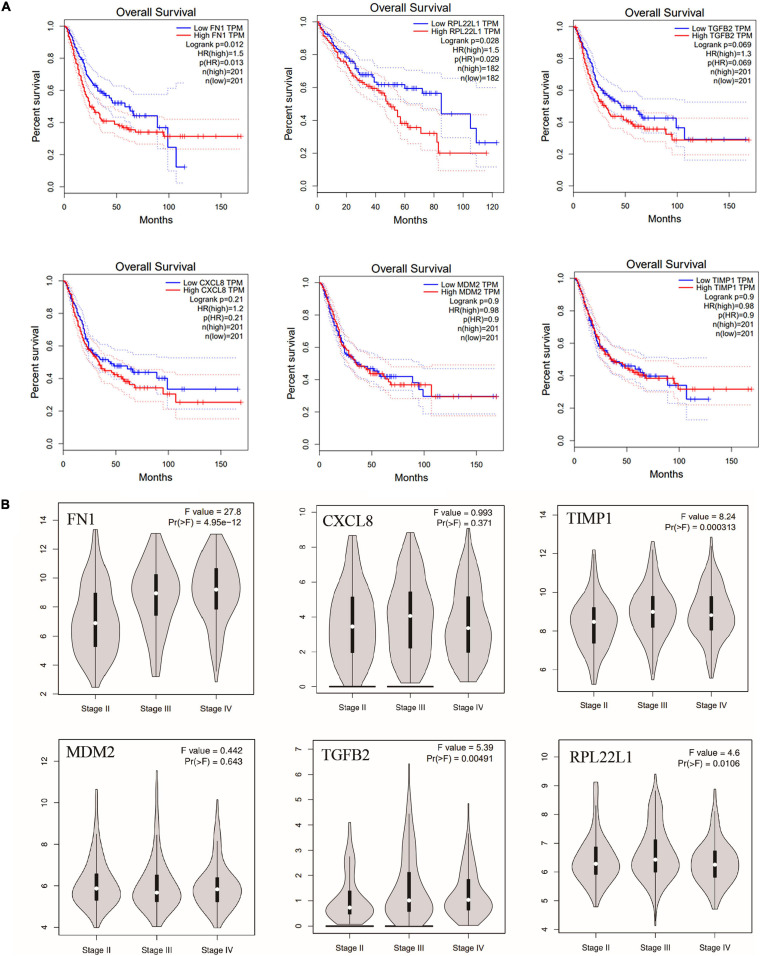
**(A)** Overall survival of the six hub genes in bladder cancer based on TCGA database, including FN1, MDM2, TGFB2, CXCL8, TIMP1, and RPL22L1. OS: overall survival. **(B)** The effect of six hub genes on bladder cancer stage.

To further investigate whether hub genes have an impact on the prognosis of bladder cancer, we performed survival analysis of CXCL8, MDM2, TGFB2, FN1, TIMP1, and RPL22L1, and their impact on tumor stage using TCGA database. Interestingly, overall survival (OS) of patients with bladder cancer with high FN1 expression was significantly lower than that with low expression (*P* = 0.012), while the OS of bladder cancer patients with low RPL22L1 expression was significantly lower than that of patients with high expression (*P* = 0.034) (Figure 7A). The other four genes had no significant effect on the prognosis of bladder cancer patients. The six hub genes also have an impact on bladder cancer stage. The expression of FN1, TIMP1, TGFB2, and RPL22L1 in Stage II was significantly lower than that in Stage III (*P* < 0.05), while the other two genes were not statistically different in stage (Figure 7B).

## Discussion

SnRNAs are a class of non-coding RNAs whose length ranges from 100 to 215 nucleotides in mammals, mainly including U1, U2, U3, U4, U5, U6, and U11 genes. SnRNAs have been present in the nucleus of mammalian cells, and together with more than 40 intranuclear proteins form RNA spliceosomes (Dvinge et al., 2019; Suzuki et al., 2019), which catalyze the maturation of precursor mRNAs in mammals, thereby affecting gene expression and leading to proliferation or apoptosis of cancer cells. We found that small nuclear RNAs mediate the formation of long-range chromatin interactions, of which U11 (RNU11) may be the most significant small nuclear RNA. To explore the gene expression changes and its potential effects mediated by U11 snRNA in bladder cancer cells, U11 snRNA knockout and overexpressed cell lines were constructed and further used to analyze the gene expression changes by RNA sequencing. Interestingly, we found that both up-regulated genes in the U11 overexpression group and down-regulated genes in the U11 knockout group were mainly enriched in cancer-related pathways, such as NF-kappa B signaling pathway, TNF signaling pathway and Bladder cancer. Protein interaction network analysis predicted that CXC chemokine family (CXCL2, CXCL3, CXCL6, and CXCL8) were hub genes. Further alternative splicing analysis also found that both the U11 knockout group and the U11 overexpression group caused alternative splicing events in genes with different expression, including some genes in the PI3K-Akt signaling pathway, such as FN1 and FGF1 genes, and other oncogenes, such as TGFB2, TIMP1, and MDM2. Our results suggest that U11 may affect the expression of cancer-related genes and be implicated in bladder carcinogenesis by affecting alternative splicing.

NF-kappa B is a heterodimer composed mainly of p65 and P50 proteins, and its function is to induce the transcription factors of inflammatory cytokines and anti-apoptotic proteins. In most cells, NF-kappa B mediates cell survival signals and protects cells from apoptosis (Jung and Dritschilo, 2001). Increasing evidence suggests that activation of NF-kappa B is associated with apoptosis, expression of angiogenic proteins, and carcinogenesis due to its fundamental effects on the dedifferentiation and proliferation of malignant tumor cells (Dorai and Aggarwal, 2004; Umezawa, 2006). Related studies have found that NF-kappa B activation is associated with urogenital cancers, such as prostate cancer and renal cell carcinoma (Oya et al., 2003; Ross et al., 2004; Domingo-Domenech et al., 2005). Similarly, in bladder tumors, the effect of NF-kappa B activation on tumorigenesis has also been reported (Levidou et al., 2008) and in our study, both up-regulated genes in the U11 overexpression group and down-regulated genes in the U11 knockout group were mainly enriched in the NF-kappa B signaling pathway.

PI3K-Akt pathway is a downstream signal transduction pathway of various cytokines and growth factors, which is involved in regulating cell proliferation and apoptosis (Lim and Counter, 2005; Bleau et al., 2009). PI3K belongs to the phospholipid kinase family and can be activated by many extracellular factors to participate in the cellular response. Activated PI3K phosphorylates PIP2–PIP3, thereby activating its downstream target kinase Akt. Activated Akt is ectopic from the cell membrane to the nucleus and cytoplasm. It activates or inhibits downstream target proteins, further promotes cell proliferation, apoptosis and energy metabolism, and is closely related to tumorigenesis and development (Franke et al., 2003). It was found that PI3K-Akt signaling pathway plays an important role in the occurrence, development and progression of malignant tumors, and the activation of Akt is closely related to the proliferation, migration and invasion of tumor cells (Roncolato et al., 2019; Ma et al., 2020). In this study, after knocking out U11, intersecting genes with significant differential alternative splicing and abnormal expression were mainly enriched in the PI3K-Akt signaling pathway.

Among them, fibronectin (FN) on the PI3K-Akt signaling pathway is a high molecular weight extracellular matrix glycoprotein with a molecular weight of 440 kDa. Its molecular structure contains a variety of domains, which can selectively bind to a variety of macromolecules in the extracellular matrix, such as collagen, heparin, fibrin and cell surface receptors, and play a crucial role in cell adhesion, migration, growth, differentiation and other cell overgrowth (Oya et al., 2003; Li et al., 2019). FN1 is a member of the FN family and plays a variety of biological functions in tumors, atherosclerosis, arthritis, and other diseases (Castelletti et al., 2008). Recent studies have found that FN1 is an important regulator promoting the formation and development of a variety of cancer cells, such as glioblastoma, laryngeal cancer and cutaneous squamous cell carcinoma (Jerhammar et al., 2010; Liao et al., 2018). In breast cancer, FN1 activates specific matrix metalloproteinases to promote breast cancer cell invasion and metastasis (Qian et al., 2011). It has been reported that the combination of miR-200c and FN1 can effectively inhibit the development of endometrial cancer in terms of FN1 expression in endometrial cancer cells (Howe et al., 2011). MiR-200c inhibits the expression of FNI, significantly reduces cell proliferation, and inhibits migration and invasion, suggesting that the expression of FN1 is a good indicator of the state of cancer cells. FN1 affects proliferation, senescence and apoptosis of human glioma cells through PI3K-AKT signaling pathway (Liao et al., 2018). Down-regulation of FN1 can inhibit proliferation, migration and invasion, thereby inhibiting the occurrence of colorectal cancer (Cai et al., 2018). Interestingly, we found that the expression of FN1 increased significantly after the knockout of U11, and FN1 underwent several meaningful alternative splicing events, including three alternative 3′ splicing site (A3SS) events, one mutually exclusive exon event, and four exon skipping events. Although we examined three typical exons skipping of FN1 (EDA, EDB, and IIICS) as previously reported (Lopez-Mejia et al., 2013), no remarkably alternative splice events were detected in FN1 gene. Due to the numerous exons of FN1 and thus generating multiple alternative splicing events, prominent bands of these AS events were extremely challenged to detect using conventional PCR. More advanced testing technologies and further versus nested PCR experiments should be conducted to detect the multiple alternative splicing events.

Chemokines essentially belong to a class of small molecule proteins, whose initial role is mainly to participate in the directional chemotaxis of leukocytes to inflammatory sites. The role of chemokines and their receptors in the process of tumorigenesis and development cannot be ignored increasingly. A large number of studies have shown that the regularity of malignant tumor cell metastasis is similar to that of chemokine migration during inflammatory cell metastasis (Ha et al., 2017). CXCL8 is an important member of the chemokine family. It was first discovered by Yoshimura in 1987 in the culture supernatant of human peripheral blood mononuclear cells stimulated with lipopolysaccharide (Yoshimura et al., 1987) and formally named IL-8/NAP (IL-8 NAP neutrophil active peptide) in 1988. At present, studies have confirmed that CXCL8 is highly expressed in thyroid tumors, ovarian cancer, liver cancer, prostate cancer and many other tumors, and its role is mainly reflected in: accelerating the growth of tumor cells, enhancing the motility of tumor cells, changing the local environment of tumors and inhibiting the immune system to play a role, and ultimately making tumor cells invade and metastasize in distant areas (Liu J. et al., 2016). In this study, we found that CXCL8 expression increased significantly after overexpression of U11, while decreased significantly after knockout of U11. Taking the intersection of the differential genes in the knockout and overexpression groups and predicting by protein interaction network analysis, we found that the chemokine family was the hub gene group, especially CXCL8. It can be seen that the expression of U11 affects the expression of the chemokine family, especially CXCL8. These results showed that the overexpression of U11 could promote the expression of chemokines, thereby promoting cell proliferation and tumor metastasis (Liu Q. et al., 2016). However, in alternative splicing analysis after knockout or overexpression of U11, we did not find significant splicing events in CXCL8, suggesting that the change in expression of U11 to CXCL8 may not be through the regulation of gene splicing. In summary, our results predict that U11 plays an important role in the regulation of chemokine expression in bladder cancer cells, but the specific mechanism is unknown.

Murine double minute 2 is a tumor protein that is highly expressed in tumors. In cancer cells, MDM2 proteins help to modify biological programs, enhance growth-promoting signals, and reduce apoptotic signals. P53 protein is a very important tumor suppressor and plays an important role in regulating cell cycle, apoptosis, DNA damage repair, angiogenesis, cell metabolism and aging (Prives, 1998; Gupta et al., 2019). In more than half of human tumors, the p53 gene is mutated or deleted, while in the remaining human tumors, there is wild-type p53, whose function is also effectively inhibited by MDM2. E3 ubiquitin ligase MDM2 is an important inhibitor of p53, which can block the transcriptional function of p53, promote the transfer of p53 from the nucleus to the cytoplasm, and ubiquitinate and degrade p53 (Brooks and Gu, 2006). In this study, we found that after overexpression of U11, MDM2 expression increased significantly, and MDM2 had a meaningful mutually exclusive exon and an exon skipping event. MDM2 was well-known as the most critical negative regulator of p53 pathway, whether the alternative splicing events of MDM2 directly or indirectly regulated by U11 deserved to be further investigated.

In summary, we found that U11 may alter gene expression by affecting the PI3K-Akt signaling pathway and NF-kappa B signaling pathway. U11 may be involved in the regulation of gene expression in bladder cancer cells, which may provide a novel biomarker for clinical diagnosis and treatment of bladder cancer.

## Materials and Methods

### Cell Lines and Cell Culture

T24 bladder cancer cell line was purchased from Cell Bank of Shanghai Institute of Cell Biology (Shanghai, China). T24-FL and T24-SLT cell lines were gifts from Dr. Gordon Hager (NIH, United States). All cells were cultured in F12 Medium (Gibco, China) supplemented with penicillin, streptomycin and 10% FBS (Gibco, Australia). All cell lines were maintained at 37°C in a humidified atmosphere containing 5% CO_2_.

### Design of sgRNA and Construction of Its Expression Vector

Using an online CRISPR design tool, two sgRNAs targeting the U11 region were designed by selecting the sgRNA sequences with high scores. The sequence is as follows:

SG1-F: 5′-CACC GCTGTCGTGAGTGGCACACGT-3′

SG1-R: 5′-AAAC ACGTGTGCCACTCACGACAGC-3′

SG2-F: 5′-CACC GCAGCTGGTGATCGTTGGTCC-3′

SG2-F: 5′-AAAC GGACCAACGATCACCAGCTGC-3′

Sequencing primers were designed with the location of sgRNA as the center. The sgRNA expression vector was cloned into pX330 all in one vector by the *Bbs*I digestion site so that the vector could express CAS9 protein and corresponding sgRNA.

### Cell Transfection

1.5 × 10^5^ T24 cells/well were plated on 24-well plates and transfected after 12–16 h (500 ng for each of the two sgRNA vectors; sgRNA vector was used as the control group). After 6–8 h, the liquid was changed. 48 h after transfection, 2 ug/ml Puro was added into the fresh medium for screening for 48 h. Cells were then grown in a fresh medium at 37°C in a humidified incubator containing 5c/o CO_2_ and collected 24 h later. The cells were subjected to genome extraction, and the other part of the cells were cloned in 96-well plates.

### Knockout of Small Nuclear RNA U11 in T24 Bladder Cancer Cells Using CRISPR/Cas9 Gene-Editing Technology

Genomic DNA was extracted with Tiangen Genome Extraction Kit and PCR was performed with sequencing primers. Three monoclonal cell lines were selected, and total RNA was extracted by the Tiangen RNA extraction kit for reverse transcription. Subsequently, the expression of U11 in wild-type and U11-knockout cells was detected by fluorescence quantitative PCR, and the knockout efficiency of U11 was identified. The monoclonal cell lines with the highest knockout efficiency were selected to construct the U11 knockout model.

### Construction of Stable Overexpressing U11 Bladder Cancer T24 Cells by pcDNA-U11 Recombinant Plasmid Transfection

RNA from T24 cells was extracted and reverse transcribed into cDNA. Using this cDNA as a template, the U11 gene sequence was amplified with specific PCR primers. The U11 fragment and pcDNA3 were digested by *Hin*dIII and *Kpn*I restriction endonucleases. The product was ligated by T4 DNA ligase and transformed into E. coli DH5α cells. The plasmid identified by sequencing was named pcDNA-U11. After transfection, identification, and monoclonal selection, the culture was expanded.

### Cell Proliferation Assay

T24^WT^ and T24^U11–KO^ cells in logarithmic growth phase were digested and seeded into 96-well plates at the concentration of 3,500 cells per well. For the MTT assay, cells were cultured for 0, 24, 48, 72, and 96 h, respectively and then 10 ul MTT (5 mg/mL) was added into each well for another 4 h at 37°C. MTT solution was then removed, and MTT formazan dissolved in 100 μL dimethyl sulfoxide (DMSO) for detection of the absorbance at 490 nm.

### Immunofluorescence

To detect expression of CBs at cellular levels, IF localization was conducted according to standard procedures. First, cells were fixed with 4% paraformaldehyde for 20 min at room temperature and permeabilized with 0.2% Triton X-100 for 10 min on ice. Then, cells were washed three times with PBS. Subsequently, cells were incubated with anti-coilin antibody (Cat# 10967-1-AP, Proteintech, United States) for 1 h, and followed by incubation with secondary antibody (Goat Anti-Mouse IgG, DyLight 488). Cells were then counterstained with DAPI after washing three times with PBS. Multicolor imaging was performed and captured utilizing an IX70 microscope at 20× magnification (Olympus, Japan).

### mRNA Library Construction and Sequencing

A total of 12 samples were taken for RNA sequencing, including 6 overexpressed U11 samples (sample64-smaple69), 2 knockout U11 samples (sample118, sample120), and 4 control samples (sample63, sample70, sample117, and sample119). Total RNA was extracted from the control group, U11 knockout bladder cancer cells, and U11 overexpressing bladder cancer cells cultured *in vitro*. The ribosome RNA was removed by ribosome RNA depletion kit and then reverse transcribed into cDNA for second-strand synthesis. dsDNA is interrupted by ultrasound to grow uniform fragments. The fragments were flattened, 3′A bases were generated, and the adaptor was ligated to complete the construction of the RNA-seq high-throughput sequencing library. High-throughput sequencing via Illumina HiSeq2000 platform. All operations were performed by Shanghai WUXI NEXTCODE. Sequencing was performed using the Illumina system, following the protocol provided by Illumina, with 2 × 150 paired-end sequencing.

### The Analysis of Alternative Splicing

After comparing the data, the file is converted to bam format using Samtools. Then alternative splice analysis was performed using rMATS 4.0.2. rMATS is a software for differential alternative splice analysis of RNA-seq data. The rMATS statistical model was used to quantify the expression of alternative splice events in different samples, and then the *P*-value was calculated by the Likelihood Ratio Test to represent the differences in LncLevel (Inclusion Level) between the two groups of samples. There are five kinds of alternative splice events recognized by rMATS, respectively skipped exon (SE), alternative 5 splice site (A5SS), A3SS, mutually exclusive exons (MXE), retained intron (RI) (Shen et al., 2014). The detailed results of alternative splicing in T24 U11-KI and T24 U11-KO cell lines are presented in Supplementary Materials.

### RNA Extraction, PCR, and DNA Agarose GEL Electrophoresis

Total RNA was extracted from the control group, U11 knockout bladder cancer cells, and U11 overexpressing bladder cancer cells cultured *in vitro* using Trizol Reagent (Invitrogen), and reverse-transcribed to cDNA using PrimeSciptTM RT reagent Kit with Gdna Eraser (Takara, China) following the manufacturer’s instructions. Primer sequence were displayed in Table 1.

**TABLE 1:** Primers used for PCR.

Gene	Forward primer 5′∼3′	Reverse primer 5′∼3′
TIMP1 Full-length	CCCTAGCGTGGACATTTATC	AAGGTGACGGGACTGGAAG
TIMP1-1	CCCTAGCGTGGACATTTATC	GGTATAAGGTGGTCTGGTTG
TIMP1-2	ACTTCCACAGGTCCCACAAC	AAGGTGACGGGACTGGAAG
TIMP1-3	CTTCTGGCATCCTGTTGTTG	GGTATAAGGTGGTCTGGTTG
GAPDH	GTGAACCATGAGAAGTAT GACAAC	CATGAGTCCTTCCACGATACC
snRNA U11	AGATAGGTAATACGACTCAC TATAG	TTAACCCTCACTAAAGG GAAGAA
	GGAAAAAGGGCTTCTGTC GTGAGTG	AGGGCGCCGGGACC

PCR was performed in 20 ul reactions containing 10 ul 2× ES Taq MasterMix (CW BIO, China), 3.4 ul H_2_O, 0.8 ul of each gene-specific primer and 5 ul cDNA. Reaction conditions were 30 cycles of 94°C for 2 min, 60°C for 30s and 72°C for 30s. PCR products were separated by 3c/o gel electrophoresis. Then Image Quant LAS 500 was used for exposure.

### Data Processing and Bioinformatics Analysis

Sequencing data is Illumina raw data of RNA-seq. Fastqc is used to evaluate the quality of raw data. Fastp is used for data pre-processing, including removing adapter components and effectively correcting lower quality bases. After fastp treatment, Fastqc detects data quality again and obtains qualified clean data. Clean data were aligned to the reference genome hg38 using Hisat2, gene expression was obtained by Stringtie, and differential genes were finally obtained by the Deseq2 R package (|Fold change| ≥ 1.5 and *P* < 0.05). Gene ontology (GO) and The Kyoto Encyclopedia of Genes and Genomes (KEGG) pathway enrichment analysis were used for DEGs using the clusterProfiler R package, and significant enrichment was defined as *P* < 0.05. Cytoscape was used to construct the U11 regulatory network. The prognostic analysis of core genes was performed using GEPIA based on the TCGA database.

## Data Availability Statement

The datasets presented in this study can be found in the Gene Expression Omnibus database (GEO) under accession number: GSE171744.

## Author Contributions

QW and ZT conceived the study. YX, ZW, and XW wrote the manuscript. YW, ST, CF, and LP analyzed the results. QL and YT edited the manuscript. All authors contributed to the article and approved the submitted version.

## Conflict of Interest

The authors declare that the research was conducted in the absence of any commercial or financial relationships that could be construed as a potential conflict of interest.

## References

[B1] Andrade L. E. Chan E. K. Raska I. Peebles C. L. Roos G. Tan E. M. (1991). Human autoantibody to a novel protein of the nuclear coiled body: immunological characterization and cDNA cloning of p80-coilin. *J. Exp. Med.* 173 1407–1419. 10.1084/jem.173.6.1407 2033369 PMC2190846

[B2] Bleau A. M. Hambardzumyan D. Ozawa T. Fomchenko E. I. Huse J. T. Brennan C. W. (2009). PTEN/PI3K/Akt pathway regulates the side population phenotype and ABCG2 activity in glioma tumor stem-like cells. *Cell Stem Cell* 4 226–235. 10.1016/j.stem.2009.01.007 19265662 PMC3688060

[B3] Bray F. Ferlay J. Soerjomataram I. Siegel R. L. Torre L. A. Jemal A. (2018). Global cancer statistics 2018: GLOBOCAN estimates of incidence and mortality worldwide for 36 cancers in 185 countries. *CA Cancer J. Clin.* 68 394–424. 10.3322/caac.21492 30207593

[B4] Brooks C. L. Gu W. (2006). p53 ubiquitination: Mdm2 and beyond. *Mol. Cell* 21 307–315. 10.1016/j.molcel.2006.01.020 16455486 PMC3737769

[B5] Cai X. Liu C. Zhang T. N. Zhu Y. W. Dong X. Xue P. (2018). Down-regulation of FN1 inhibits colorectal carcinogenesis by suppressing proliferation, migration, and invasion. *J. Cell Biochem.* 119 4717–4728. 10.1002/jcb.26651 29274284

[B6] Castelletti F. Donadelli R. Banterla F. Hildebrandt F. Zipfel P. F. Bresin E. (2008). Mutations in FN1 cause glomerulopathy with fibronectin deposits. *Proc. Natl. Acad. Sci. U.S.A.* 105 2538–2543. 10.1073/pnas.0707730105 18268355 PMC2268172

[B7] Domingo-Domenech J. Mellado B. Ferrer B. Truan D. Codony-Servat J. Sauleda S. (2005). Activation of nuclear factor-kappaB in human prostate carcinogenesis and association to biochemical relapse. *Br. J. Cancer* 93 1285–1294. 10.1038/sj.bjc.6602851 16278667 PMC2361509

[B8] Dorai T. Aggarwal B. B. (2004). Role of chemopreventive agents in cancer therapy. *Cancer Lett.* 215 129–140. 10.1016/j.canlet.2004.07.013 15488631

[B9] Dvinge H. Guenthoer J. Porter P. L. Bradley R. K. (2019). RNA components of the spliceosome regulate tissue- and cancer-specific alternative splicing. *Genome Res.* 29 1591–1604. 10.1101/gr.246678.118 31434678 PMC6771400

[B10] Feng R. M. Zong Y. N. Cao S. M. Xu R. H. (2019). Current cancer situation in China: good or bad news from the 2018 Global Cancer Statistics? *Cancer Commun.* 39:22. 10.1186/s40880-019-0368-6 31030667 PMC6487510

[B11] Franke T. F. Hornik C. P. Segev L. Shostak G. A. Sugimoto C. (2003). PI3K/Akt and apoptosis: size matters. *Oncogene* 22 8983–8998. 10.1038/sj.onc.1207115 14663477

[B12] Gupta A. Behl T. Heer H. R. Deshmukh R. Sharma P. L. (2019). Mdm2-P53 interaction inhibitor with cisplatin enhances apoptosis in colon and prostate cancer cells In-Vitro. *Asian Pac. J. Cancer Prev.* 20 3341–3351. 10.31557/APJCP.2019.20.11.3341 31759358 PMC7062994

[B13] Ha H. Debnath B. Neamati N. (2017). Role of the CXCL8-CXCR1/2 Axis in Cancer and inflammatory diseases. *Theranostics* 7 1543–1588. 10.7150/thno.15625 28529637 PMC5436513

[B14] Hearst S. M. Gilder A. S. Negi S. S. Davis M. D. George E. M. Whittom A. A. (2009). Cajal-body formation correlates with differential coilin phosphorylation in primary and transformed cell lines. *J. Cell Sci.* 122(Pt 11), 1872–1881. 10.1242/jcs.044040 19435804 PMC2684838

[B15] Hebert M. D. (2010). Phosphorylation and the Cajal body: modification in search of function. *Arch. Biochem. Biophys.* 496 69–76. 10.1016/j.abb.2010.02.012 20193656 PMC2850958

[B16] Hebert M. D. (2013). Signals controlling Cajal body assembly and function. *Int. J. Biochem. Cell Biol.* 45 1314–1317. 10.1016/j.biocel.2013.03.019 23583661 PMC3683375

[B17] Howe E. N. Cochrane D. R. Richer J. K. (2011). Targets of miR-200c mediate suppression of cell motility and anoikis resistance. *Breast Cancer Res.* 13:R45. 10.1186/bcr2867 21501518 PMC3219208

[B18] Jeppesen D. K. Nawrocki A. Jensen S. G. Thorsen K. Whitehead B. Howard K. A. (2014). Quantitative proteomics of fractionated membrane and lumen exosome proteins from isogenic metastatic and nonmetastatic bladder cancer cells reveal differential expression of EMT factors. *Proteomics* 14 699–712. 10.1002/pmic.201300452 24376083

[B19] Jerhammar F. Ceder R. Garvin S. Grénman R. Grafström R. C. Roberg K. (2010). Fibronectin 1 is a potential biomarker for radioresistance in head and neck squamous cell carcinoma. *Cancer Biol. Ther.* 10 1244–1251. 10.4161/cbt.10.12.13432 20930522

[B20] Jung M. Dritschilo A. (2001). NF-kappa B signaling pathway as a target for human tumor radiosensitization. *Semin. Radiat. Oncol.* 11 346–351. 10.1053/srao.2001.26034 11677659

[B21] Levidou G. Saetta A. A. Korkolopoulou P. Papanastasiou P. Gioti K. Pavlopoulos P. (2008). Clinical significance of nuclear factor (NF)-kappaB levels in urothelial carcinoma of the urinary bladder. *Virchows. Arch.* 452 295–304. 10.1007/s00428-007-0560-y 18188593

[B22] Li B. Shen W. Peng H. Li Y. Chen F. Zheng L. (2019). Fibronectin 1 promotes melanoma proliferation and metastasis by inhibiting apoptosis and regulating EMT. *Onco Targets Ther.* 12 3207–3221. 10.2147/OTT.S195703 31118673 PMC6503329

[B23] Liang Z. Mou Q. Pan Z. Zhang Q. Gao G. Cao Y. (2019). Identification of candidate diagnostic and prognostic biomarkers for human prostate cancer: RPL22L1 and RPS21. *Med. Oncol.* 36:56. 10.1007/s12032-019-1283-z 31089825

[B24] Liao Y. X. Zhang Z. P. Zhao J. Liu J. P. (2018). Effects of fibronectin 1 on cell proliferation, senescence and apoptosis of human glioma cells through the PI3K/AKT signaling pathway. *Cell Physiol. Biochem.* 48 1382–1396. 10.1159/000492096 30048971

[B25] Lim K. H. Counter C. M. (2005). Reduction in the requirement of oncogenic Ras signaling to activation of PI3K/AKT pathway during tumor maintenance. *Cancer Cell* 8 381–392. 10.1016/j.ccr.2005.10.014 16286246

[B26] Liu J. Xu R. Zhao X. (2016). [Mechanisms for effect of osthole on inhibiting the growth and invasion of bladder cancer cells]. *Zhong Nan Da Xue Xue Bao Yi Xue Ban* 41 345–352. 10.11817/j.issn.1672-7347.2016.04.002 27241143

[B27] Liu Q. Li A. Tian Y. Wu J. D. Liu Y. Li T. (2016). The CXCL8-CXCR1/2 pathways in cancer. *Cytokine Growth Factor Rev.* 31 61–71. 10.1016/j.cytogfr.2016.08.002 27578214 PMC6142815

[B28] Lopez-Mejia I. C. De Toledo M. Della Seta F. Fafet P. Rebouissou C. Deleuze V. (2013). Tissue-specific and SRSF1-dependent splicing of fibronectin, a matrix protein that controls host cell invasion. *Mol. Biol. Cell* 24 3164–3176. 10.1091/mbc.E13-03-0142 23966470 PMC3806663

[B29] Lui L. Lowe T. (2013). Small nucleolar RNAs and RNA-guided post-transcriptional modification. *Essays Biochem*. 54, 53–77. 10.1042/bse0540053 23829527

[B30] Ma Z. Yu R. Zhu Q. Sun L. Jian L. Wang X. (2020). CXCL16/CXCR6 axis promotes bleomycin-induced fibrotic process in MRC-5 cells via the PI3K/AKT/FOXO3a pathway. *Int. Immunopharmacol.* 81:106035. 10.1016/j.intimp.2019.106035 31753588

[B31] Machyna M. Heyn P. Neugebauer K. M. (2013). Cajal bodies: where form meets function. *Wiley Interdiscip. Rev. RNA* 4 17–34. 10.1002/wrna.1139 23042601

[B32] Nicholson B. E. Frierson H. F. Conaway M. R. Seraj J. M. Harding M. A. Hampton G. M. (2004). Profiling the evolution of human metastatic bladder cancer. *Cancer Res.* 64 7813–7821. 10.1158/0008-5472.Can-04-0826 15520187

[B33] Oya M. Takayanagi A. Horiguchi A. Mizuno R. Ohtsubo M. Marumo K. (2003). Increased nuclear factor-kappa B activation is related to the tumor development of renal cell carcinoma. *Carcinogenesis* 24 377–384. 10.1093/carcin/24.3.377 12663495

[B34] Ploeg M. Aben K. K. Kiemeney L. A. (2009). The present and future burden of urinary bladder cancer in the world. *World J. Urol.* 27 289–293. 10.1007/s00345-009-0383-3 19219610 PMC2694323

[B35] Prives C. (1998). Signaling to p53: breaking the MDM2-p53 circuit. *Cell* 95 5–8. 10.1016/s0092-8674(00)81774-2 9778240

[B36] Qian P. Zuo Z. Wu Z. Meng X. Li G. Wu Z. (2011). Pivotal role of reduced let-7g expression in breast cancer invasion and metastasis. *Cancer Res.* 71 6463–6474. 10.1158/0008-5472.CAN-11-1322 21868760

[B37] Roncolato F. Lindemann K. Willson M. L. Martyn J. Mileshkin L. (2019). PI3K/AKT/mTOR inhibitors for advanced or recurrent endometrial cancer. *Cochrane Database Syst. Rev.* 10:CD012160. 10.1002/14651858.CD012160.pub2 31588998 PMC6953296

[B38] Ross J. S. Kallakury B. V. Sheehan C. E. Fisher H. A. Kaufman R. P. Jr. Kaur P. (2004). Expression of nuclear factor-kappa B and I kappa B alpha proteins in prostatic adenocarcinomas: correlation of nuclear factor-kappa B immunoreactivity with disease recurrence. *Clin. Cancer Res.* 10 2466–2472. 10.1158/1078-0432.ccr-0543-3 15073126

[B39] Salim E. I. Moore M. A. Bener A. Habib O. S. Seif-Eldin I. A. Sobue T. (2010). Cancer epidemiology in South-West Asia - past, present and future. *Asian Pac. J. Cancer Prev.* 11(Suppl. 2), 33–48. 20553067

[B40] Shen S. Park J. W. Lu Z. X. Lin L. Henry M. D. Wu Y. N. (2014). rMATS: robust and flexible detection of differential alternative splicing from replicate RNA-Seq data. *Proc. Natl. Acad. Sci. U.S.A.* 111 E5593–E5601. 10.1073/pnas.1419161111 25480548 PMC4280593

[B41] Strzelecka M. Trowitzsch S. Weber G. Lührmann R. Oates A. C. Neugebauer K. M. (2010). Coilin-dependent snRNP assembly is essential for zebrafish embryogenesis. *Nat. Struct. Mol. Biol.* 17 403–409. 10.1038/nsmb.1783 20357773

[B42] Suzuki H. Kumar S. A. Shuai S. Diaz-Navarro A. Gutierrez-Fernandez A. De Antonellis P. (2019). Recurrent noncoding U1 snRNA mutations drive cryptic splicing in SHH medulloblastoma. *Nature* 574 707–711. 10.1038/s41586-019-1650-0 31664194 PMC7141958

[B43] Umezawa K. (2006). Inhibition of tumor growth by NF-kappaB inhibitors. *Cancer Sci.* 97 990–995. 10.1111/j.1349-7006.2006.00285.x 16925581 PMC11158475

[B44] Usher P. A. Sieuwerts A. M. Bartels A. Lademann U. Nielsen H. J. Holten-Andersen L. (2007). Identification of alternatively spliced TIMP-1 mRNA in cancer cell lines and colon cancer tissue. *Mol. Oncol.* 1 205–215. 10.1016/j.molonc.2007.05.002 19383295 PMC5543896

[B45] Wang Q. Sawyer I. A. Sung M. H. Sturgill D. Shevtsov S. P. Pegoraro G. (2016). Cajal bodies are linked to genome conformation. *Nat. Commun.* 7:10966. 10.1038/ncomms10966 26997247 PMC4802181

[B46] Yoshimura T. Matsushima K. Oppenheim J. J. Leonard E. J. (1987). Neutrophil chemotactic factor produced by lipopolysaccharide (LPS)-stimulated human blood mononuclear leukocytes: partial characterization and separation from interleukin 1 (IL 1). *J. Immunol.* 139 788–793. 3298433

